# Activin a is associated with impaired myocardial glucose metabolism and left ventricular remodeling in patients with uncomplicated type 2 diabetes

**DOI:** 10.1186/1475-2840-12-150

**Published:** 2013-10-17

**Authors:** Weena JY Chen, Sabrina Greulich, Rutger W van der Meer, Luuk J Rijzewijk, Hildo J Lamb, Albert de Roos, Johannes WA Smit, Johannes A Romijn, Johannes B Ruige, Adriaan A Lammertsma, Mark Lubberink, Michaela Diamant, D Margriet Ouwens

**Affiliations:** 1 Diabetes Center, Department of Internal Medicine, VU University Medical Center, Amsterdam, the Netherlands; 2 Institute of Clinical Biochemistry and Pathobiochemistry, German Diabetes Center, Düsseldorf, Germany; 3 Department of Radiology, Leiden University Medical Center, Leiden, the Netherlands; 4 Department of General Internal Medicine 463, Radboud University Medical Center Nijmegen, Nijmegen, The Netherlands; 5 Department of Medicine, Academic Medical Center, Amsterdam, The Netherlands; 6 Department of Endocrinology, Ghent University Hospital, Ghent, Belgium; 7 Department of Radiology & Nuclear Medicine & PET Research, VU University Medical Center, Amsterdam, The Netherlands; 8 German Center for Diabetes Research (DZD), Neuherberg, Germany; 9 Present address: Department of Radiology, Oncology and Radiation Science, Section of Nuclear Medicine and PET, Uppsala University, Uppsala, Sweden

**Keywords:** Activin A, Myocardial glucose metabolism, Cardiac remodeling, Diabetic cardiomyopathy

## Abstract

**Background:**

Activin A released from epicardial adipose tissue has been linked to contractile dysfunction and insulin resistance in cardiomyocytes. This study investigated the role of activin A in clinical diabetic cardiomyopathy by assessing whether circulating activin A levels associate with cardiometabolic parameters in men with uncomplicated type 2 diabetes (T2D), and the effects of treatment with pioglitazone versus metformin on these associations.

**Methods:**

Seventy-eight men with uncomplicated T2D and fourteen healthy men with comparable age were included, in this randomized, double-blind, active comparator intervention study. All T2D men were on glimipiride monotherapy, and randomized to a 24-week intervention with either pioglitazone or metformin. Cardiac dimensions and -function were measured using magnetic resonance imaging, whilst myocardial glucose metabolism (MMRglu) was determined using [^18^F]-2-fluoro-2-deoxy-D-glucose positron emission tomography during a hyperinsulinemic-euglycemic clamp.

**Results:**

Circulating activin A levels were comparable in T2D men and controls. Activin A levels were independently inversely associated with MMRglu, and positively with left ventricular mass/volume (LVMV)-ratio in T2D men. Intervention with metformin decreased activin A levels, whereas pioglitazone did not alter activin A levels. The changes in plasma activin A levels were not correlated with the changes in MMRglu following either pioglitazone or metformin treatment. A borderline significant correlation (p = 0.051) of changes in plasma activin A levels and changes in LVMV-ratio was observed after pioglitazone treatment.

**Conclusions:**

Circulating activin A levels are associated with impaired myocardial glucose metabolism and high LVMV-ratio in patients with uncomplicated T2D, reflecting a potential detrimental role in early human diabetic cardiomyopathy.

**Trial registration number:**

Current Controlled Trials SRCTN53177482

## Background

Diabetic cardiomyopathy is a multifactorial condition characterized by an impaired cardiac function independent of coronary artery disease or hypertension [1]. Changes in myocardial substrate metabolism are described to contribute to its pathogenesis. Subtle defects in cardiac structure and function can be detected before the presence of clinically evident cardiac disease [2,3]. These defects are amendable to therapeutic interventions since beneficial effects of pioglitazone on myocardial glucose metabolism and diastolic function in patients with type 2 diabetes (T2D) have been reported [4].

Epicardial adipose tissue (EAT) is a visceral fat depot directly surrounding the myocardium [5]. Emerging evidence shows that factors secreted by EAT exert paracrine effects on cardiac metabolism and contractile function, hence, with the potential to contribute to the development of cardiovascular disease [5]. Especially activin A is of interest in this context. An enhanced release of activin A from EAT of patients with T2D impairs cardiomyocyte function *in vitro*[6]. Furthermore, the fibrotic potential of the EAT secretome has been ascribed to activin A [7].

This study aimed at investigating whether activin A impacts on the pathophysiology of diabetic cardiomyopathy. Therefore, we determined circulating activin A levels in men with uncomplicated T2D and healthy men of comparable age, evaluated the association of activin A levels with cardiac function and –metabolism, and examined the impact of intervention with pioglitazone and metformin on these associations.

## Methods

### Participants

Circulating activin A levels were determined in fasting plasma samples from participants of the previously described PIRAMID (Pioglitazone Influence on tRiglyceride Accumulation in the Myocardium in Diabetes) study [4,8]. This study included 78 T2D men, aged 45–65 years, with an HbA1c of 6.5 – 8.5 %, a body mass index (BMI) of 25–32 kg/m^2^, and a sitting blood pressure (BP) less than 150/85 mmHg. Furthermore, we included 14 healthy men of comparable age with normal glucose metabolism as determined by a 75-g oral glucose tolerance test. The T2D men were randomized in this 24-week randomized, double-blind, double-dummy with active comparator trial, to pioglitazone (15 mg once daily, titrated to 30 mg once daily after 2 weeks) or metformin (500 mg twice daily, titrated to 1000 mg twice daily) and matching placebo. The PIRAMID study was conducted at two university medical hospitals in the Netherlands (Leiden University Medical Center, Leiden, and VU University Medical Center, Amsterdam), and approved by the medical ethics committee of both centers. This study was performed in full compliance with the Declaration of Helsinki.

### Cardiac magnetic resonance imaging (MRI)

All participants underwent MRI scanning on a 1.5 Tesla whole-body MR scanner (Gyroscan ACS/NT15; Philips, Best, the Netherlands) after an overnight fast. Technical procedures were performed as described earlier [4,9]. Images were analyzed quantitatively with dedicated software (MASS and FLOW, Medis, Leiden, the Netherlands). During MRI, BP and heart rate (HR) were measured. Left ventricular (LV) mass/volume ratio (LVMV-ratio) was calculated as the ratio between LV enddiastolic mass and LV enddiastolic volume.

### Positron emission tomography (PET) protocol

All PET examinations were performed using an ECAT EXACT HR + scanner (Siemens/CTI, Knoxville, Tennessee). Myocardial metabolic rate of glucose (MMRglu) was measured during a hyperinsulinemic-euglycemic clamp procedure [10], using [^18^F]-2-fluoro-2-deoxy-D-glucose ([^18^F]-FDG). After injection of 185 MBq [^18^F]-FDG, a 60-min dynamic emission scan was acquired.

### PET image analysis

PET data were quantitatively reconstructed using filtered back projection after all appropriate corrections. To generate myocardial time-activity curves, regions of interest were defined on resliced LV short-axis (summed) [^18^F]-FDG images and subsequently projected onto the dynamic images. Regions of interest were drawn as previously described [11], and grouped for further analysis. A separate aorta ascendance region of interest was defined to generate an ^18^F-FDG image-derived input function. MMRglu was calculated by multiplying the net rate of influx constant of ^18^F-FDG, *K*_i_, by the mean plasma glucose concentration. *K*_i_ was determined using Patlak graphic analysis [12].

### Biochemical analysis

Plasma activin A levels were determined using an Enzyme Linked Immuno Sorbent Assay (R&D systems, Minneapolis, MN, USA). The lower detection limit of the assay was 3.67 pg/mL. The intra- and interassay coefficients of variation were 4.4% and 5.0%, respectively. This assay shows no significant cross-reactivity with activin B and activin C, and 0.45% cross-reactivity with Activin AB, and measures both free and follistatin-bound activin A dimers.

### Statistical analysis

Data are expressed as mean ± standard deviation of the mean or median (interquartile range) when non-normally distributed. Group differences were determined using students *t*-test for normal distributed data, and Mann–Whitney U-test for skewed distributed data. Correlation coefficients were calculated using Pearson’s correlation. Ln-transformed data were used in case of skewed distributions. Linearity of the regression models was judged based on histograms and scatter plots. Additional potential confounders were investigated by adding age, BMI, systolic BP (SBP), diastolic BP (DBP), HR, fasting glucose, and fasting triglyceride levels in the multivariate analysis of activin A with LVMV-ratio and aortic pulse wave velocity (PWV). Next, age, BMI, SBP, DBP, HR, fasting glucose, insulin, and triglyceride levels, and M-value for insulin sensitivity were added in the multivariate analysis for associations of activin A with MMRglu. Variables that changed regression coefficients by more than 10% were included in the adjusted model. Between-group comparisons were performed using ANCOVA with adjustments for intervention group and baseline values. Within-group changes from baseline were assessed using independent paired *t–*test for normal distributed data and Wilcoxon signed-ranked test for skewed distributed data. Statistical analyses were performed using SPSS software version 20.0 (IBM corporation, New York, USA). A value of *P <* 0.05 was considered as statistically significant.

## Results

### Plasma activin A levels in T2D men versus controls and its relationship with metabolic and cardiac parameters

**Figure 1:**
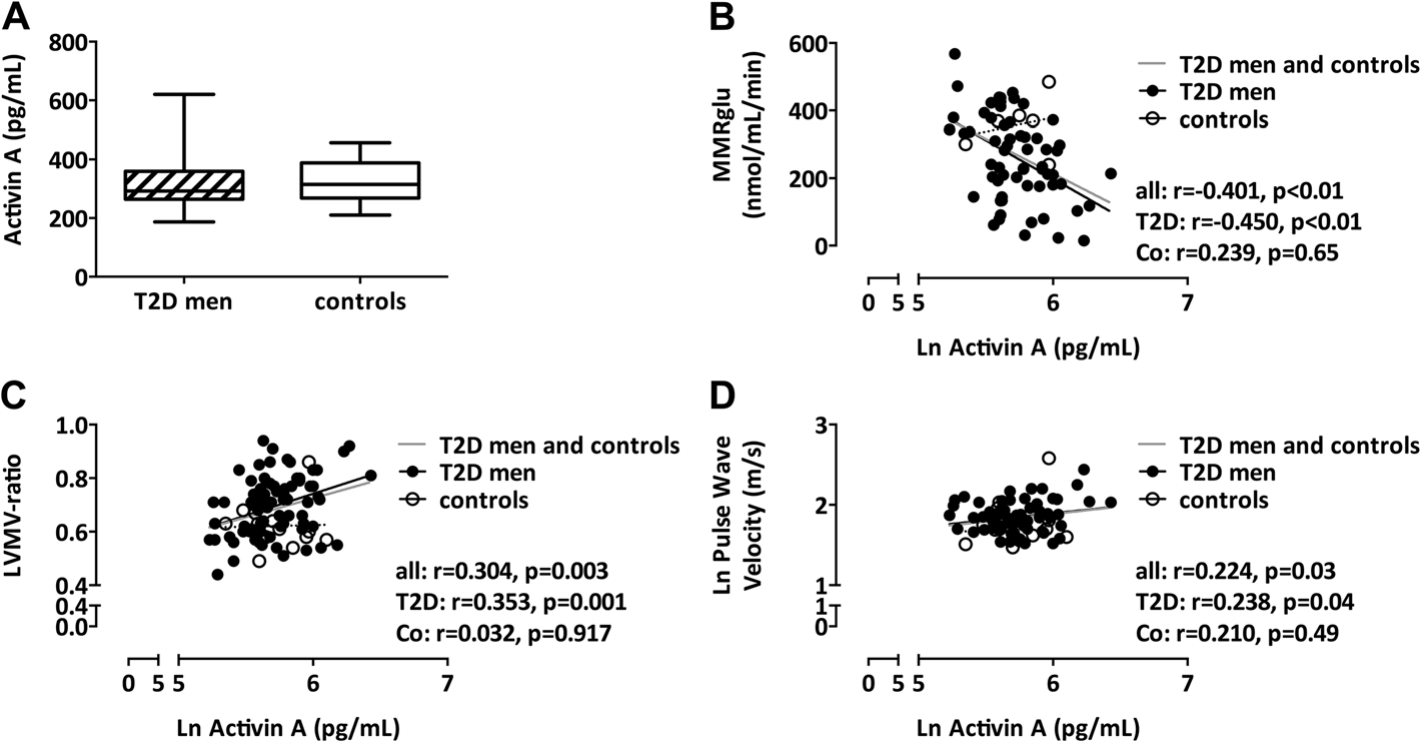
**Plasma activin A levels in men with uncomplicated type 2 diabetes versus controls. (A)** Whisker plots (median, min – max) of plasma activin A levels in 78 type 2 diabetic (T2D) men and 14 healthy control men. Differences in plasma activin A levels were analyzed using a Mann–Whitney U-test. Regression analyses showed significant inverse correlation of plasma activin A levels with myocardial metabolic rate of glucose for all subjects, T2D men and control (Co) subjects (MMRglu; **B)**, and positive correlations with left ventricular mass/volume ratio (LVMV-ratio; **C)**, and aortic pulse wave velocity (PWV; **D)** in T2D men (black dots with black regression line) and controls (white dots with dashed regression line). Grey lines represent pooled regression lines.

The anthropometric and cardiometabolic variables of the participants were described previously [13]. Importantly, T2D men had impaired metabolic control and LV diastolic dysfunction [13] (Additional file 1: Table S1). Median activin A levels were comparable between T2D men and controls (293 versus 315 pg/mL, p = 0.42; Figure 1A). Univariate regression analyses (Additional file 2: Table S2) showed inverse correlations between activin A levels and MMRglu (r = −0.450, p < 0.001; Figure 1B) and positive correlations with age (r = 0.232, p = 0.04), diabetes duration (r = 0.236, p = 0.04), triglycerides (r = 0.258, p = 0.02), SBP (r = 0.277, p = 0.01), LVMV-ratio (r = 0.353, p = 0.002; Figure 1C), and PWV (r = 0.238, p = 0.04; Figure 1D) in T2D. Notably, plasma activin A levels were not significantly correlated with fasting glucose levels (r = −0.210, p = 0.07), fasting insulin levels (r = 0.154, p = 0.18), or M-value (r = −0.190, p = 0.11), nor with diastolic function parameters (all p > 0.05; Additional file 2: Table S2). In multivariate analysis of the T2D patients, the association of activin A levels with MMRglu was not affected by adjustment for M-value (β = −0.373, p = 0.001). Age, SBP, DBP, BMI, as well as fasting glucose, insulin, or triglyceride levels did not change the regression coefficient. The association of activin A levels with LVMV-ratio remained significant after adjustment for SBP (β = 0.276, p = 0.02). However, age and SBP were confounding factors in the association of activin A levels with PWV (β = 0.151, p = 0.13). Other variables as DBP, HR, BMI, fasting glucose, or triglyceride levels did not change the regression coefficients of the association between activin A levels and either LVMV-ratio or PWV.

### Effects of pioglitazone and metformin on plasma activin A levels

**Figure 2:**
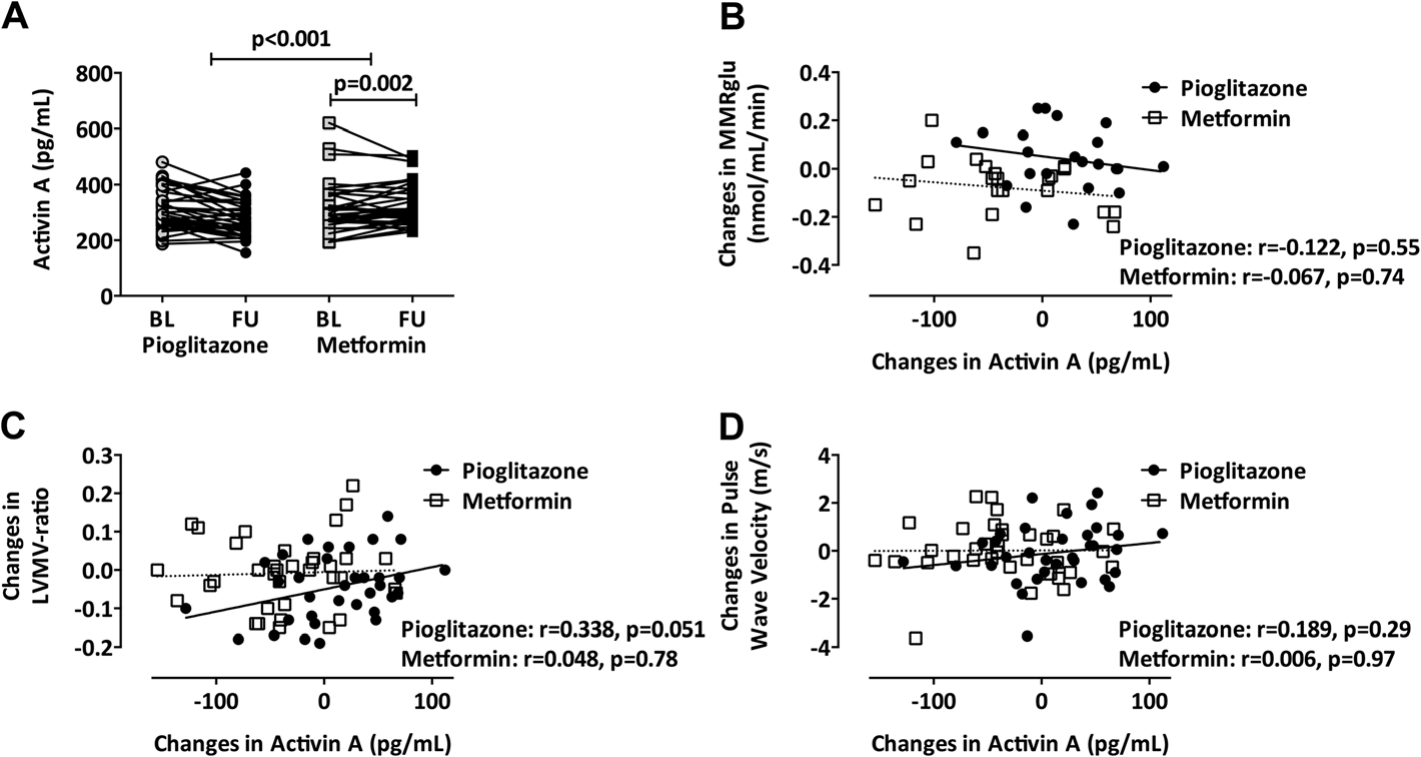
**Effects of 24-week pioglitazone versus metformin on activin A levels in type 2 diabetic men. (A)** Plasma activin A levels at baseline (BL, light grey) and at 24-weeks follow-up (FU, dark grey) after intervention with pioglitazone (dots) versus metformin (squares) in men with uncomplicated type 2 diabetes (T2D). Differences in plasma activin A levels at BL and FU in each intervention group were analyzed using Wilcoxon matched-pair signed-rank test, between-group differences were performed using linear regression analysis with adjustments for intervention group and baseline values. Pearson correlation analysis showed that changes in activin A levels were not related to changes in MMRglu after either pioglitazone (black dots, black regression line) or metformin (white squares, dashed regression line; **B)**. A marginally significant positive correlation was seen between changes in activin A levels and changes in left ventricular mass/volume ratio (LVMV-ratio) after pioglitazone, not after metformin **(C)**. Changes in activin A levels were not correlated with changes in pulse wave velocity after either pioglitazone (black dots, black regression line) or metformin (white squares, dashed regression line; **D)**.

Previously, we reported that treatment with pioglitazone or metformin improved glycemic control in both groups [4]. Furthermore, an improvement in diastolic function and myocardial glucose metabolism after pioglitazone treatment was observed as compared to metformin treatment, together with a decrease in LVMV-ratio only after pioglitazone treatment [4]. Neither pioglitazone nor metformin impacted on PWV (Additional file 3: Table S3). Treatment with pioglitazone did not change activin A levels (293 to 302 pg/mL, p = 0.13; Figure 2A). After metformin, activin A levels decreased (293 to 261 pg/mL, p = 0.002; Figure 2A). Between-group analysis showed a decrease of activin A levels after metformin versus pioglitazone, with adjustment for baseline activin A levels (p < 0.001; Figure 2A). Changes in activin A levels were correlated neither with changes in MMRglu (Figure 2B) nor with changes in PWV (Figure 2D) following treatment with either pioglitazone or metformin. A borderline significant correlation of changes in activin A levels and changes in LVMV-ratio was observed after pioglitazone (r = 0.338, p = 0.051), but not after metformin treatment (r = 0.048, p = 0.78; Figure 2C).

## Discussion

This study shows that circulating plasma activin A levels in T2D men without cardiovascular complications associate with impaired myocardial glucose metabolism, independent of M-value. Furthermore, we observed a positive association with the LVMV-ratio. Metformin treatment decreased activin A levels, while pioglitazone had no effect. Furthermore, in patients treated with pioglitazone, changes in plasma activin A levels were borderline significantly correlated with changes observed in LVMV-ratio. There was no association between changes in plasma activin A and myocardial glucose metabolism after either pioglitazone or metformin treatment. These results suggest an involvement of activin A in the pathogenesis of early cardiac derangements in T2D.

Importantly, the pathogenesis of diabetic cardiomyopathy is not yet completely unraveled. It has been proposed that myocardial dysfunction in patients with T2D is related to depletion of endothelial progenitor cells and increased oxidative stress [14]. Others demonstrated that early T2D is associated with an increase in the sarco/endoplasmic reticulum Ca^2+^-ATPase (SERCA) / phospholamban-ratio and that insulin directly stimulates SERCA expression and relaxation velocity [15]. Finally, circulating levels of osteoprotegerin and adiponectin were found to associate with cardiac abnormalities in men with uncomplicated T2D [16]. The present study identifies activin A as additional potential factor contributing to the pathogenesis of myocardial dysfunction in diabetic cardiomyopathy.

Levels of circulating activin A were in the same range as other clinical studies [17-19]. Although one study reported that high activin A levels associated with abnormal glucose regulation in patients with myocardial infarction though without known T2D [20], we as well as others did not find altered activin A levels between patients with (uncomplicated) T2D and controls [19,21,22]. Nevertheless, activin A levels tended to be higher in patients with cardiovascular disease [17]. In patients with stable and unstable angina, levels of activin A were elevated as compared to healthy controls [17]. Also in T2D patients with coronary artery disease higher levels of activin A were found as compared to T2D patients without coronary artery disease [19]. Finally, in heart failure patients, increased activin A levels were demonstrated as compared to healthy controls [18,19].

Some studies have reported beneficial effects of activin A. Activin A is a dimeric protein consisting of two inhibin βA monomers linked by a single disulfide bond. In mice, maternal diabetes induces cardiac malformations in the embryos by downregulation of inhibin βA, and in association decreased activation of the post-receptor signaling components Smad2 and/or Smad3 [23,24]. Furthermore, Zhao et al. demonstrated that activin A was able to rescue myocardial cell proliferation and endocardial cell migration in mouse embryonic hearts which was suppressed by maternal diabetes [25]. Other investigators have proposed that activin A could have beneficial effects on inflammation and atherogenesis, and that high activin A levels reflect a counteracting anti-inflammatory and anti-oxidative response [19,20]. The present findings do not support this, but are corroborated by our earlier studies in which we showed that activin A derived from EAT from T2D patients impairs cardiomyocyte insulin sensitivity by inhibiting the insulin-mediated phosphorylation of Akt through induction of microRNA-143 [6,26].

The association of activin A with LVMV-ratio indicates that this factor is involved in early myocardial remodeling in T2D as well, as LVMV-ratio is one of the features of isolated LV diastolic dysfunction [7,13,27]. Importantly, this is supported by our results showing that changes in activin A levels after only pioglitazone were borderline positively correlated with changes in LVMV-ratio. Although an *in vitro* study on monocytes reported no effect of pioglitazone on activin A release [21], it should be noted that the key effector for pioglitazone, PPARγ, is predominantly expressed in adipose tissue. Furthermore, expression is activin A is ubiquitous [6,7,28,29], and high expression levels have been reported in human adipocyte progenitor cells [29]. During differentiation into adipocytes activin A expression is downregulated, and inhibition of the activin A signaling has been found to promote human adipogenesis [29]. Based on these observations, one may speculate that pioglitazone improves adipocyte differentiation and adipose tissue quality, thereby lowering activin A production by mature adipocytes. Interestingly, a recent report directly linked activin A released from EAT to the development of cardiac fibrosis [7]. This raises the possibility that the reduction of the LVMV-ratio in pioglitazone-treated patients may result from local effects of pioglitazone on epicardial adipocytes rather than a systemic effect on circulating activin A levels. Accordingly, we could recently demonstrate that activin A release is increased in EAT from patients with T2D [6,26]. This fat depot is not separated by a fascia from the underlying tissue and shares the blood supply with the myocardium. Consequently, activin A released from EAT can directly affect cardiac function in a paracrine fashion.

The current findings do not completely exclude the possibility that the effects of pioglitazone on the LVMV-ratio result from an improved left ventricular diastolic function in this treatment group. In support of this notion is that metformin treatment did not affect cardiac function, including the LVMV-ratio [4], despite lowering circulating activin A levels. An *in vitro* study on monocytes also reported a downregulation of activin A release following metformin exposure [21]. However, the absence of a beneficial effect of metformin on determinants of left ventricular diastolic function may prevent beneficial effects on left ventricular hypertrophy despite lower circulating activin A levels.

Another issue that should be considered is that the expression and release of activin A is not confined to EAT. Rather activin A is expressed by a large number of cells and tissues, including (epicardial) adipose tissue [6,7,28,29]. Because the circulating levels of activin A were not different between controls and patients with T2D, it seems likely that the amount of activin A found in the circulation is also derived from these other sources. Nevertheless, both our previous *in vitro* studies [6,26] and the present clinical study support the notion that activin A participates in the development of cardiac abnormalities in patients with T2D.

### Limitations

This study has several limitations. First, the number of control subjects examined in this study was relatively small. Consequently, additional studies remain required to confirm the lack of association between activin A and cardiometabolic parameters in subjects without T2D. Second, our study was conducted in males. Although this may limit generalization of our findings, it should be noted that multiple studies showed that gender is not a confounding factor for levels of activin A levels in the circulation [18,19,22,30,31].

## Conclusions

This is the first report showing independent relationships of plasma activin A levels with both impaired myocardial glucose metabolism and increased LVMV-ratios in T2D without known cardiac complications. As the pathogenesis of diabetic cardiomyopathy is not fully understood, the present data provide evidence for a potential role of activin A in the development of early diabetic cardiomyopathy. However, the data should be interpreted with caution, as further studies are warranted to identify the exact role of activin A in cardiac remodeling in diabetic cardiomyopathy.

## Abbreviations

BMI: Body mass index; BP: Blood pressure; DBP: Diastolic blood pressure; EAT: Epicardial adipose tissue; [18F]-FDG: [^18^F]-2-fluoro-2-deoxy-D-glucose; HR: Heart rate; LV: Left ventricular; LVMV-ratio: Left ventricular mass/volume-ratio; MMRglu: Myocardial metabolic rate of glucose; MRI: Magnetic resonance imaging; M-value: Whole body insulin sensitivity; PET: Positron emission tomography; PWV: Pulse wave velocity; SBP: Systolic blood pressure; T2D: Type 2 diabetes.

## Competing interests

MD is a consultant and speaker for Eli Lilly and Company, Novo Nordisk and Merck, Sharp and Dohme, and a consultant for Sanofi-Aventis, Astra-Zeneca/BMS and Novartis Pharma. Through MD the VU University Medical Center in Amsterdam has received research grants from Amylin Pharmaceuticals Inc, Eli Lilly and Company, Novo Nordisk, Merck, Sharp and Dohme, Novartis and Takeda. The other authors have no conflicts of interest to report.

## Authors’ contributions

WJYC interpreted the data, performed the statistical analysis and drafted the manuscript. SG contributed in the analysis and interpretation of the data, and drafted the manuscript. RWM participated in acquisition of the data, coordinated the study, interpretation of the data and critically reviewed the manuscript. LJR participated in acquisition of the data, coordinated the study, interpretation of the data and critically reviewed the manuscript. HJL contributed to the design of the study, interpretation of the data, and critically reviewed the manuscript. AR participated in analysis and interpretation of the data, and critically reviewed the manuscript. JWAS contributed to the design of the study, interpretation of the data, and critically reviewed the manuscript. JAR contributed to the design of the study, interpretation of the data, and critically reviewed the manuscript. JBR participated in the interpretation of the data, and critically reviewed the manuscript. AAL participated in analysis and interpretation of the data, and critically reviewed the manuscript. ML participated in analysis and interpretation of the data, and critically reviewed the manuscript. MD contributed to the design of the study, interpretation of the data, and critically reviewed the manuscript. All authors read and approved the final manuscript. DMO contributed to the analysis and interpretation of the data, and critically reviewed the manuscript. All authors read and approved the final manuscript.

## Supplementary Material

Additional file 1: Table S1 Clinical, Biochemical and Cardiovascular Characteristics. Click here for file

Additional file 2: Table S2 Correlations between circulating activin A levels and anthropometric, biochemical parameters, myocardial glucose metabolism, and cardiac dimensions and –function in men with type 2 diabetes. Click here for file

Additional file 3: Table S3 Effects of Pioglitazone versus Metformin on Clinical, Biochemical and Cardiovascular Characteristics. Click here for file
