# Supplementary material for: Activin a is associated with impaired myocardial glucose metabolism and left ventricular remodeling in patients with uncomplicated type 2 diabetes
Source: Cardiovasc Diabetol. 2013 Oct 17;12:150. doi: 10.1186/1475-2840-12-150 (PMC4015886; doi:10.1186/1475-2840-12-150)
Supplement: Additional file 1: Table S1 — Clinical, Biochemical and Cardiovascular Characteristics. [file 1475-2840-12-150-S1.docx]

**Additional file 1: Table S1. Clinical, Biochemical and Cardiovascular Characteristics**

|  | **Men with T2D**  **(n=78)** | **Controls**  **(n=14)** |
| --- | --- | --- |
| **Baseline characteristics, insulin sensitivity** |  |  |
| Age, years^‡^ | 56.5 ± 5.6 | 54.5 ± 7.1 |
| Diabetes duration, years^‡^ | 4 (2-6) | NA |
| Use of antihypertensive medication, n (%)^‡^ | 34 (44) | NA |
| Statin use, n (%)^‡^ | 38 (49) | NA |
| Aspirin use, n (%) | 0 (0) | NA |
| BMI, kg/m^2‡^ | 28.7 ± 3.5 | 27.0 ± 2.5^**^ |
| M-value, mg/kg.min^‡^ | 2.7 (1.6-4.2) | 8.1 (7.4-10.0)^***^ |
| **Plasma parameters** |  |  |
| Fasting plasma glucose, mmol/L^‡^ | 8.3 (7.1-10.0) | 5.3 (5.0-5.6)^***^ |
| Fasting plasma insulin, pmol/L^‡^ | 64 (36-92) | 28 (19-33)^***^ |
| HbA1c, %^‡^ | 7.1 ± 1.0 | 5.3 ± 0.2^***^ |
| Total cholesterol, mmol/L^‡^ | 4.7 ± 1.0 | 5.3 ± 0.7^***^ |
| HDL-cholesterol, mmol/L^‡^ | 1.1 (0.9-1.3) | 1.4 (1.3-1.6)^***^ |
| Triglycerides, mmol/L^‡^ | 1.5 (1.0-2.2) | 0.8 (0.7-1.1)^***^ |
| Plasma non-esterified fatty acids, mmol/L^‡^ | 0.50 (0.40-0.62) | 0.46 (0.37-0.52) |
| **Myocardial glucose metabolism** |  |  |
| Myocardial metabolic rate of glucose, nmol/mL/min^‡^ | 260 ± 128 | 348 ± 154^*^ |
| **Hemodynamic parameters, cardiac dimensions and function** | | |
| Systolic blood pressure, mm Hg^‡^ | 128 ± 12 | 118 ± 11^***^ |
| Diastolic blood pressure, mm Hg^‡^ | 76 ± 7 | 72 ± 8^*^ |
| Heart rate, beats/min^‡^ | 64 (60-70) | 52 (51-62)^***^ |
| Rate pressure product, (beats/min).mm Hg^‡^ | 8345 ± 1457 | 6684 ± 1441^***^ |
| LV mass, gram^‡^ | 107 ± 17 | 111 ± 24 |
| LVMV-ratio, gram/mL | 0.70 ± 0.11 | 0.63 ± 0.09^*^ |
| LV end systolic volume, mL^‡^ | 59 (52-71) | 72 (63-82)^***^ |
| Stroke volume, mL^‡^ | 94 ± 16 | 107 ± 23^**^ |
| Ejection fraction, %^‡^ | 60 ± 6 | 59 ± 4 |
| Pulse wave velocity, m/s | 6.7 (5.5-7.0) | 5.4 (4.9-6.2)^**^ |
| E peak filling rate, mL/s^‡^ | 417 ± 84 | 503 ± 112^***^ |
| E deceleration peak, mL/s^2^.10^-3‡^ | 3.4 (2.9-4.0) | 4.7 (3.1-5.2)^**^ |
| E deceleration mean, ml/s^2^.10^-3^ | 2.3 ± 0.7 | 2.7 ± 0.8^*^ |
| E/A peak ratio^‡^ | 1.0 ± 0.3 | 1.3 ± 0.4^*^ |

Data are mean ± SD or median (interquartile range). P-values for differences between variables were calculated using the students *t*-test in case of normally distributed data, or the Mann-Whitney U-test in case of non-Gaussian distributions data. ***, indicates *P*<0.001; **, *P*<0.01; *, *P*<0.05. T2D, type 2 diabetes; BMI, body mass index; M-value, whole body insulin sensitivity; HbA1c, glycosylated hemoglobin; HDL, high-density lipoprotein; LV, left ventricular; LVMV-ratio, left ventricular mass/volume ratio; E, early diastolic filling phase; A, diastolic atrial contraction. ^‡^Adapted from Rijzewijk et al. 2009. J Am Coll Cardiol 54:1524-32.
