# Supplementary material for: Activin a is associated with impaired myocardial glucose metabolism and left ventricular remodeling in patients with uncomplicated type 2 diabetes
Source: Cardiovasc Diabetol. 2013 Oct 17;12:150. doi: 10.1186/1475-2840-12-150 (PMC4015886; doi:10.1186/1475-2840-12-150)
Supplement: Additional file 2: Table S2 — Correlations between circulating activin A levels and anthropometric, biochemical parameters, myocardial glucose metabolism, and cardiac dimensions and –function in men with type 2 diabetes. [file 1475-2840-12-150-S2.docx]

**Additional file 2: Table S2. Correlations between circulating activin A levels and anthropometric, biochemical parameters, myocardial glucose metabolism, and cardiac dimensions and –function in men with type 2 diabetes**

|  | **Pearson’s r** | **P-value** |
| --- | --- | --- |
| **Baseline characteristics, insulin sensitivity** |  | |
| Age, years | 0.232 | 0.04 |
| Diabetes duration, years | 0.236 | 0.04 |
| Use of antihypertensive medication, n (%) | -0.230 | 0.83 |
| Statin use, n (%) | -0.005 | 0.96 |
| BMI, kg/m^2^ | 0.064 | 0.58 |
| M-value, mg/kg.min | -0.190 | 0.11 |
| **Plasma parameters** |  |  |
| Fasting plasma glucose, mmol/L | -0.210 | 0.07 |
| Fasting plasma insulin, pmol/L | 0.154 | 0.18 |
| HbA1c, % | -0.037 | 0.75 |
| Total cholesterol, mmol/L | 0.172 | 0.13 |
| HDL-cholesterol, mmol/L | 0.093 | 0.42 |
| Triglycerides, mmol/L | 0.258 | 0.02 |
| Plasma non-esterified fatty acids, mmol/L | 0.164 | 0.16 |
| **Myocardial glucose metabolism** |  |  |
| Myocardial metabolic rate of glucose, nmol/mL/min | -0.450 | <0.001 |
| **Hemodynamic parameters, cardiac dimensions and function** | | |
| Systolic blood pressure, mm Hg | 0.277 | 0.01 |
| Diastolic blood pressure, mm Hg | 0.208 | 0.07 |
| Heart rate, beats/min | -0.055 | 0.63 |
| Rate pressure product, (beats/min).mm Hg | 0.100 | 0.38 |
| LV mass, gram | 0.152 | 0.18 |
| LVMV-ratio, gram/mL | 0.353 | 0.002 |
| LV end systolic volume, mL | -0.171 | 0.13 |
| Stroke volume, mL | -0.161 | 0.16 |
| Ejection fraction, % | 0.043 | 0.71 |
| Pulse wave velocity, m/s | 0.238 | 0.04 |
| E peak filling rate, mL/s | -0.151 | 0.19 |
| E deceleration peak, mL/s^2^.10^-3^ | -0.112 | 0.33 |
| E deceleration mean, ml/s^2^.10^-3^ | -0.120 | 0.29 |
| E/A peak ratio | -0.079 | 0.49 |

BMI, body mass index; M-value, whole body insulin sensitivity; HbA1c, glycosylated hemoglobin; HDL, high-density lipoprotein; LV, left ventricular; LVMV-ratio, left ventricular mass/volume ratio; E, early diastolic filling phase; A, diastolic atrial contraction.
