# Supplementary material for: Activin a is associated with impaired myocardial glucose metabolism and left ventricular remodeling in patients with uncomplicated type 2 diabetes
Source: Cardiovasc Diabetol. 2013 Oct 17;12:150. doi: 10.1186/1475-2840-12-150 (PMC4015886; doi:10.1186/1475-2840-12-150)
Supplement: Additional file 3: Table S3 — Effects of Pioglitazone versus Metformin on Clinical, Biochemical and Cardiovascular Characteristics. [file 1475-2840-12-150-S3.docx]

**Additional file 3: Table S3. Effects of Pioglitazone versus Metformin on Clinical, Biochemical and Cardiovascular Characteristics**

|  | **Pioglitazone (n=34)** | | | | **Metformin (n=37)** | | | **P-value (between groups at Follow-up)** |
| --- | --- | --- | --- | --- | --- | --- | --- | --- |
|  | **Baseline** | | **Follow-up** | | **Baseline** | **Follow-up** | |  |
| **Clinical and Biochemical Parameters** | | | | | | | | |
| BMI, kg/m^2^ | 28.0 ± 2.8 | | | 28.9 ± 3.4^*^ | 29.1 ± 3.7 | | 28.9 ± 4.1 | <0.001 |
| M-value, mg/kg.min | 2.9 ± 1.8 | | | 3.4 ± 1.7^*^ | 3.2 ± 1.8 | | 3.6 ±2.3 | 0.03 |
| Fasting plasma glucose, mmol/L^‡^ | 8.4 (7.2 – 10.3) | | | 7.6 (6.7-9.4)^**^ | 8.2 (6.8-9.1) | | 6.8 (5.8-7.4)^**^ | 0.14 |
| Fasting plasma insulin, pmol/L^‡^ | 58 (38-83) | | | 49 (34-70) | 80 (31-99) | | 59 (32-98) | 0.15 |
| HbA1c, %^‡^ | 7.1 ± 1.0 | | | 6.5 ± 0.8^***^ | 7.0 ± 0.8 | | 6.3 ± 0.6^***^ | 0.15 |
| Total cholesterol, mmol/L^‡^ | 4.5 ± 0.9 | | | 4.6 ± 1.0 | 4.9 ± 0.9 | | 4.5 ±0.2^**^ | 0.04 |
| HDL-cholesterol, mmol/L^‡^ | 1.1 (0.90 – 1.3) | | | 1.2 (1.0-1.5)^**^ | 1.1 (0.9-1.4) | | 1.0 (0.9-1.3) | 0.009 |
| Triglycerides, mmol/L^‡^ | 1.4 (1.0 – 2.2) | | | 1.4 (0.9 - 2.3) | 1.5 (0.9-2.1) | | 1.7 (0.9-2.3) | 0.60 |
| Plasma non-esterified fatty acids, mmol/L^‡^ | 0.45 (0.41-0.59) | | | 0.46 (0.34-0.57) | 0.53 (0.39-0.77) | | 0.49 (0.39-0.56) | 0.93 |
| **Myocardial glucose metabolism** | | | | | | | | |
| Myocardial metabolic rate of glucose, nmol/mL/min^‡^ | 259 ± 123 | 333 ± 102^*^ | | | 262 ± 141 | 193 ± 109^**^ | | <0.001 |
| **Hemodynamic parameters, cardiac dimensions and function** | | | | | | | | |
| Systolic blood pressure, mm Hg^‡^ | 130 ± 12 | | 125 ± 12^*^ | | 126 ± 11 | 121 ± 10^*^ | | 0.49 |
| Diastolic blood pressure, mm Hg^‡^ | 77 ± 7 | | 74 ± 8 | | 74 ± 7 | 73 ± 7 | | 0.97 |
| Heart rate, beats/min^‡^ | 65 ± 9 | | 63 ± 7 | | 65 ± 8 | 64 ± 8 | | 0.90 |
| Rate pressure product, (beats/min).mm Hg^‡^ | 8508 ± 1492 | | 7853 ± 1137^*^ | | 8206 ± 1307 | 7744 ± 1173^**^ | | 0.77 |
| LV mass, gram^‡^ | 108 ± 14 | | 105 ± 16 | | 107 ± 19 | 103 ± 18 | | 0.54 |
| LVMV-ratio, gram/mL | 0.68 ± 0.11 | | 0.64 ± 0.08^**^ | | 0.71 ± 0.11 | 0.70 ± 0.11 | | 0.009 |
| LV ejection fraction, %^‡^ | 59 ± 6 | | 60 ± 5 | | 61 ± 5 | 60 ± 5 | | 0.53 |
| Pulse wave velocity, m/s | 6.3 (5.6-7.4) | | 6.2 (5.7-7.3) | | 5.9 (5.4-6.6) | 6.1 (5.4-7.1) | | 0.59 |
| E peak filling rate, mL/s^‡^ | 422 ± 89 | | 440 ± 81 | | 409 ± 85 | 407 ± 78 | | 0.05 |
| E deceleration peak, mL/s^2^.10^-3‡^ | 3.5 ± 1.1 | | 3.8 ± 1.1^*^ | | 3.5 ± 1.0 | 3.5 ± 1.0 | | 0.11 |
| E deceleration mean, ml/s^2^.10^-3‡^ | 2.3 ± 0.7 | | 2.4 ± 0.6 | | 2.3 ± 0.7 | 2.2 ± 0.7 | | 0.06 |
| E/A peak ratio^‡^ | 1.1 ± 0.3 | | 1.1 ± 0.3 | | 1.0 ± 0.2 | 1.0 ± 0.2 | | 0.35 |

Data are mean ± SD or median (interquartile range).P-values for within-groups were calculated using the paired *t*-test in case of normally distributed data, or the Wilcoxon signed-rank test in case of non-Gaussian distributions data. ***, indicates *P*<0.001; **, *P*<0.01; *, *P*<0.05 for within-group changes from baseline. BMI, body mass index; M-value, whole body insulin sensitivity; HbA1c, glycosylated hemoglobin; HDL, high-density lipoprotein; LV, left ventricular; LVMV-ratio, left ventricular mass/volume ratio; E, early diastolic filling phase; A, diastolic atrial contraction.

^‡^Adapted from van der Meer et al. 2009. Circulation 119:2069-2077.
